# Influence of Sulfate-Reducing Bacteria on the Corrosion Behavior of High Strength Steel EQ70 under Cathodic Polarization

**DOI:** 10.1371/journal.pone.0162315

**Published:** 2016-09-07

**Authors:** Fang Guan, Xiaofan Zhai, Jizhou Duan, Meixia Zhang, Baorong Hou

**Affiliations:** 1 Marine Corrosion and Protection Centre, Institute of Oceanology, Chinese Academy of Sciences, No. 7 Nanhai Road, Qingdao, 266071, China; 2 University of Chinese Academy of Sciences, Beijing, 100049, China; University of Akron, UNITED STATES

## Abstract

Certain species of sulfate-reducing bacteria (SRB) use cathodes as electron donors for metabolism, and this electron transfer process may influence the proper protection potential choice for structures. The interaction between SRB and polarized electrodes had been the focus of numerous investigations. In this paper, the impact of cathodic protection (CP) on *Desulfovibrio caledoniens* metabolic activity and its influence on highs trength steel EQ70 were studied by bacterial analyses and electrochemical measurements. The results showed that EQ70 under -0.85 V_SCE_ CP had a higher corrosion rate than that without CP, while EQ70 with -1.05 V_SCE_ had a lower corrosion rate. The enhanced SRB metabolic activity at -0.85 V_SCE_ was most probably caused by the direct electron transfer from the electrode polarized at -0.85 V_SCE_. This direct electron transfer pathway was unavailable in -1.05 V_SCE_. In addition, the application of cathodic protection led to the transformation of sulfide rusts into carbonates rusts. These observations have been employed to provide updated recommendations for the optimum CP potential for steel structures in the presence of SRB.

## Introduction

**Fig 1:**
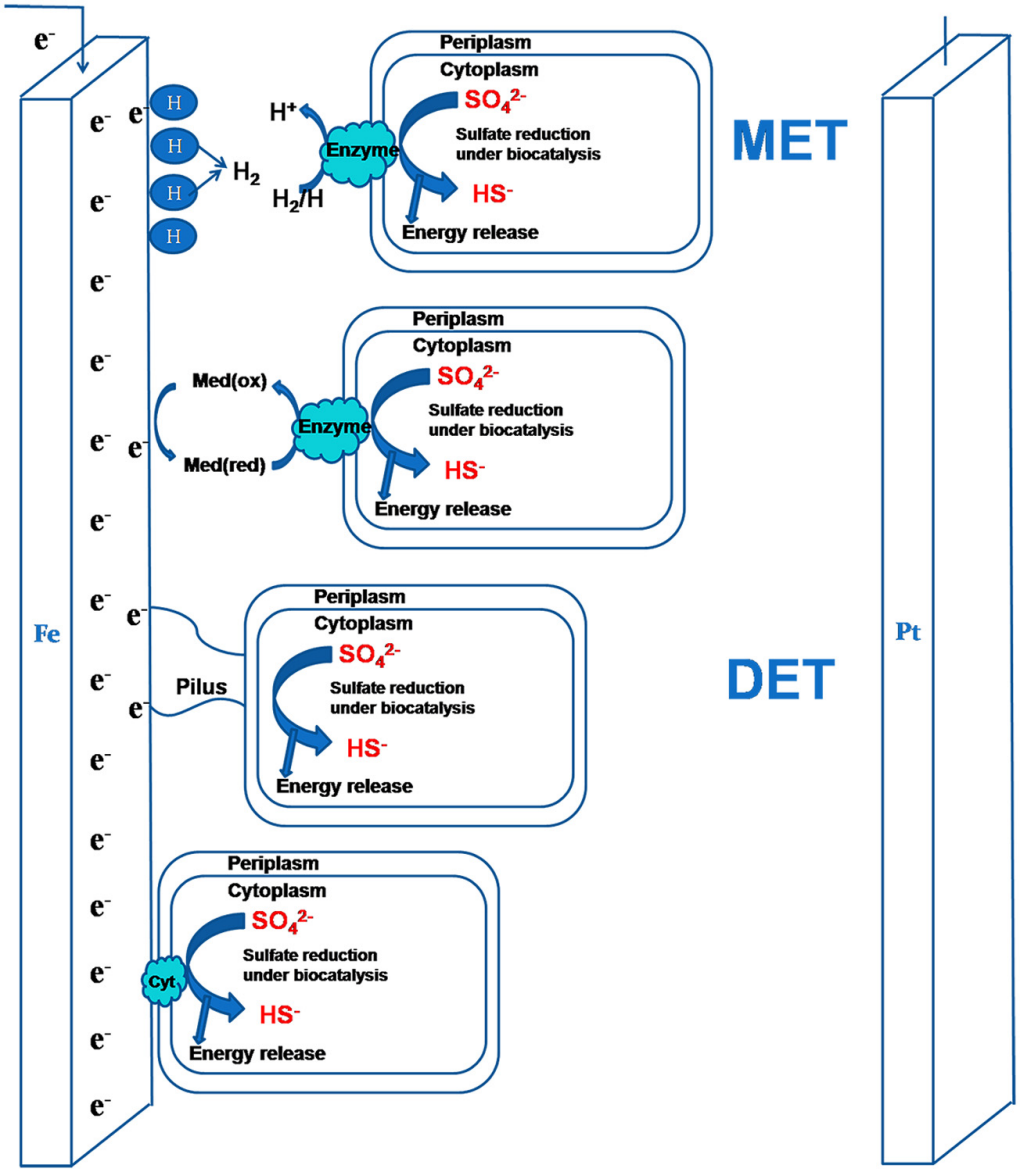
Schematic representation of the interaction between cathodic protection and activity of sulfate reducing bacteria.

Sulfate-reducing bacteria (SRB) are a major bacterial group involved in microbiologically influenced corrosion (MIC) [1, 2]. The role of SRB in iron corrosion can be divided into direct corrosion and indirect corrosion. Indirect corrosion is the chemical attack by hydrogen sulfide [3, 4] or other acidic organisms [5, 6]. Direct corrosion uses the consumption of cathodic hydrogen and iron-derived electron transfers [7]. However, when outer polarization is applied, the interaction between polarized electrode and SRB metabolic activity should not be ignored. The electron transfer pathways between SRB and polarized electrodes can be viewed from two categories: mediated electron transport (MET) that utilizes redox-active chemical mediators and direct electron transfer (DET) that relies on specific protein-based structures or nanowires of bacteria (Fig 1).

Dissolved hydrogen is an electron mediator used by SRB as an electron carrier. Studies found that cathodically produced hydrogen at proper potential values facilitated the growth of hydrogenase-positive bacteria [8–10]. The SRB biofilm could obtain electrons from the cathodically polarized graphite electrode to form hydrogen [11]. Under the protection potential applied to the system, the H^+^ ions move to the metal surface, where they are adsorbed and reduced as shown in Eq 3. The generated protons (H) on steel surface formed a hydrogen film. They are then oxidized by hydrogenase enzyme to reduce sulfates for SRB metabolic activity [12]. This process becomes cyclic and causes pH increase. Several studies have shown that some SRB strains could get electrons by hydrogen depolarization, for example, *D*. *caledoniensis* [11]was reported could obtain electrons from polarized electrodes with hydrogen evolution and oxidation; *Desulfitobacterium* [13] could catalyze H_2_ production without mediators at cathode potentials lower than -0.7 V_SHE_; and *Geobactersul furreducens*, a well-known current producing micro-organism, could produce electricity using hydrogen produced at polarized electrodes [14]. The electron transfer from polarized electrodes provided a new method of corrosion. Other electron transfer mediators like riboflavin and flavin adenine dinucleotide [15] were found to play an important role in electron transfer and accelerate SRB corrosion[16]. *Dechlorinating* bacteria, for example, are known to contain cyanocobalamin (vitamin B_12_) and other redox active co-factors [17].

DET means that SRB gets electrons directly from cathodic polarized electrodes by specific protein-based structures (such as c-type cytochromes [18]) or pilus-like conductive nanowires. Studies have indicated that the energy metabolism of SRB is closely associated with a c-type cytochrome network that connects multiple periplasmic hydrogenases and formate dehydrogenases [19]. Sherar [20] indicated that in microcosm slacking carbon sources, SRB formed nanowires to extract nutrients from the corroding steel. A similar result was found in *Desulfovibrio vulg*aris biofilm [21]. It is possible that pili nanowires are also involved in the electrons transfer.

$$Fe\to Fe^{2+}+2e^{-}$$ (1)

$$H_{2}O\to OH^{-}+H^{+}$$ (2)

$$H^{+}+e^{-}\to \left[H\right]$$ (3)

$$SO_{4}^{2-}+8\left[H\right]+H^{+}\to HS^{-}+4H_{2}O$$ (4)

$$Fe^{2+}+S^{2-}\to FeS$$ (5)

$$4Fe+SO_{4}^{2-}+4H_{2}O\to FeS+3Fe{\left(OH\right)}_{2}+2OH^{-}$$ (6)

overall reaction DET

$$SO_{4}^{2-}+8e^{-}+9H^{+}\to HS^{-}+4H_{2}O$$ (7)

$$Fe-2e^{-}\to Fe^{2+}$$ (8)

$$4Fe+SO_{4}^{2-}+4H_{2}O\to FeS+3Fe{\left(OH\right)}_{2}+2OH^{-}$$ (9)

In addition, deposited ferrous sulfide formed during the corrosion process could also mediate electron flow between metal and microorganism cells [22–24]. The electron transfer between SRB and polarized electrodes changes the corrosion behavior of steels in the presence of SRB.

Studies have shown that amore negative potential was needed to prevent steel structures from crevice corrosion with SRB than that under abiotic conditions [25, 26]. In practice, a potential of -0.85 V_SCE_ is sufficient in most cases to protect iron structures in aerated seawater. However, this potential must be lowered to -0.95 V_SCE_ due to the possible presence of SRB that usually predominates in anaerobic environments beneath biofilms. It was found that the optimum cathodic polarization potential (OCPP) of carbon steel in sea mud shifted negatively in presence of SRB (-1.03 V_SCE_), and more negative potential is needed. Moreover, the biofilm structural characteristics and inorganic deposits were different from those formed on steel without protection. Thus when applying CP to a metallic structure, it is necessary to carry out different studies to determine the physical, chemical, and microbiological characteristics of the surrounding environment [27, 28]. This will help to establish the proper potential required to achieve effective protection.

As a useful engineering material, high strength steel finds widespread use in a wide range of seawater application. Niu [29] investigated the effect of microstructure on crack tip opening displacement of high strength steel EQ70. The concentration of H absorbed by the high strength steel was higher at more cathodic potentials and significantly increased when SRB were present [30, 31]. Special precautions should be taken when the environment contains SRB [32]. The interactive effects between CP and microbiological corrosion of high strength steel structures immersed in seawater have received limited attention in the literature [31, 32].

Here, we focused on the interaction between SRB metabolic activity and cathodic potential and its influence on high strength steel EQ70. The effect of cathodic polarized electrode on SRB was evaluated. Further, the effects of the applied potential and SRB on the corrosion behavior of EQ70 were investigated.

## Experimental

### Bacteria and culture media

*Desulfovibrio caledoniensis*, a species of SRB, isolated from the rust layers of carbon steel near Qingdao City was chosen and a recent study had showed that it could obtain electrons directly from polarized electrodes [11]. SRB cultures were inoculated in sterile Postgate’s C (PGC) medium containing: 0.5 g K_2_HPO_4_; 1.0 g NH_4_Cl; 0.06 g CaCl_2_∙6H_2_O; 0.06 g MgSO_4_∙7H_2_O; 6 ml 70% sodium lactate; 1.0 g yeast cream; 0.3 g sodium citrate and 1 L aged seawater from Qingdao offshore area. The medium was degassed by purging with high-purity nitrogen for 20 min and autoclaved at 121°C. Five-day-old bacteria (1% v/v) were injected into the electrochemical reactors, where the working electrode was suspended for biocorrosion experiments. The SO_4_^2-^ was determined to study the growth of SRB in the assay using a DX-120 ion chromatography system (Dionex Corporation) with a carbonate-selective anion-exchange column (Dionex AS9-HC).

### Electrode materials

The EQ70 high strength steel coupons used in the present study are composed of the following: 1.05 Mn, 0.52 Si, 0.02 S, 0.02 P, 0.18 C, 0.98 Cr, with the balance Fe. Cylindrical EQ70 specimens (diameter = 15 mm) were used as working electrodes for electrochemical test. To create these electrodes, a copper wire was connected to the reverse of each coupon and mounted in epoxy resin. The surface was polished using P120 to P3000 grit SiC papers and cleaned with ethanol. The working electrodes were subjected to ultraviolet light for at least 30 min before use to insure no contamination by other bacteria.

### Instrumentation and experimentation

Electrochemical measurements were carried out using athree electrode arrangement. A platinum plate with an area of 4 cm^2^ was used as a counter electrode and a saturated calomel electrode (SCE) as the reference electrode. Electrochemical tests were carried out using a Solartron 1287/1260 electrochemical analyzer. A Luggin capillary was used to decrease the IR drop.

**Fig 2:**
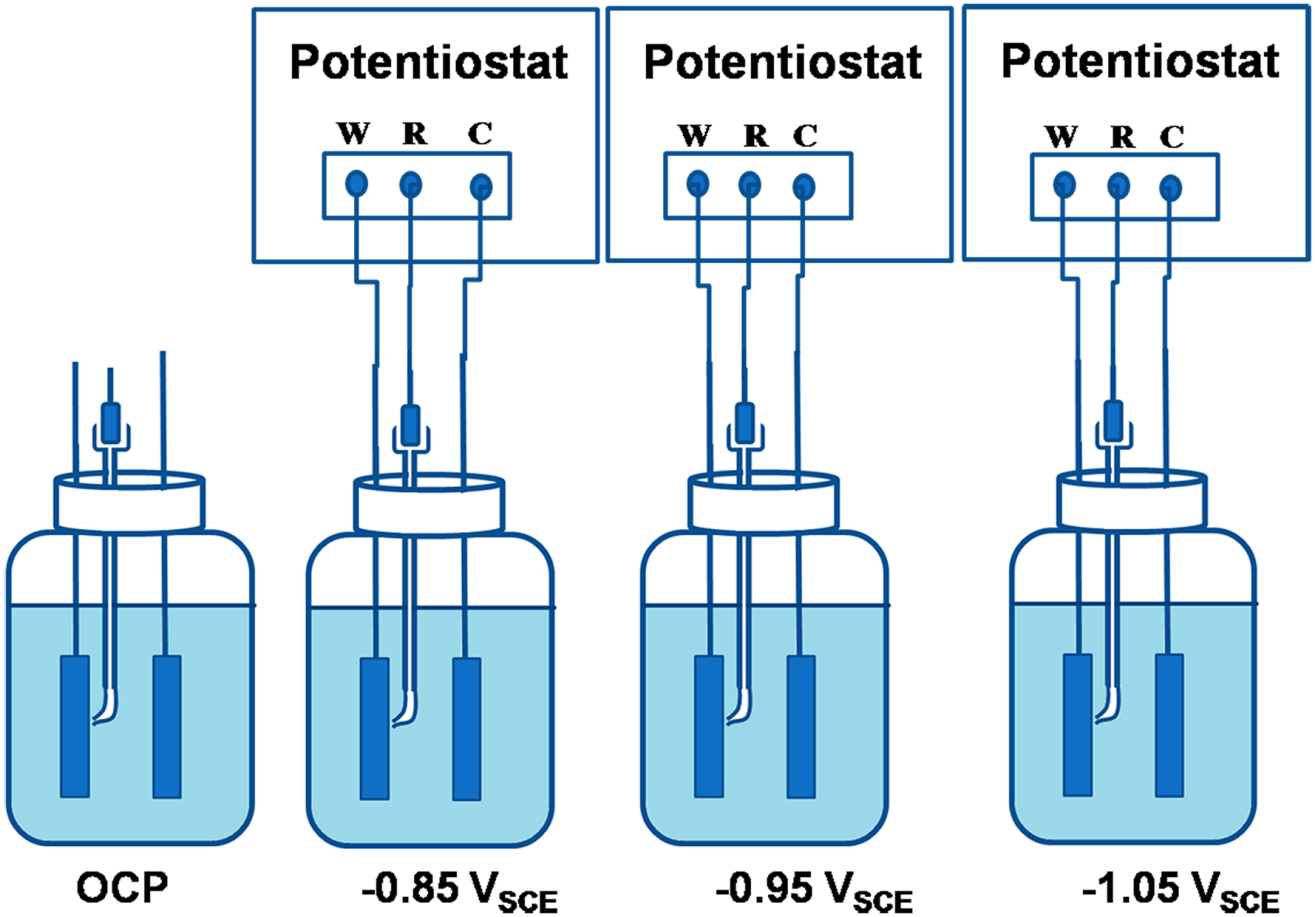
Schematic illustration of experimental cells.

The specimens were stabilized for about 15days, applying separate cathodic polarization potential of -0.85 V_SCE_, -0.95 V_SCE_, -1.05 V_SCE_; and no polarization as control (Fig 2). The applied potential was removed before electrical impedance spectroscopy (EIS) measurement, until their open-circuit potential (OCP stabilized. The EIS was measured using a 10 mV sinusoidal signal with frequencies ranging from 100 kHz to 10 mHz. On the 15th day, EIS was also measured at polarization potentials of -0.85 V_SCE_, -0.95 V_SCE_, -1.05 V_SCE_. The results were analyzed using Princeton ZSimpWin version 3.21 software.

Polarization curves were determined potentiodynamically at a scan rate of 0.5 mVs^-1^ after 15 days exposure to the test solution.

### Surface analysis

After immersing in SRB medium for 15days with different cathodic polarization potentials, the EQ70 coupons were taken out for SEM observations. The surface appearance of the EQ70 in the SEM was visualized after a preparation using the following procedure. Samples were fixed in 2.5% glutaraldehyde for 2 hand then dehydrated using an ethanol gradient (at 50%, 75%, 95% and100%) for 15 min. Coupons were then dried at the critical point and sputter-coated with gold prior to observation.

X-ray photoelectron spectroscopy (XPS, ESCALAB250 surface analysis system, Thermo VG, USA) was used to determine the chemical states of sulfur on the surfaces utilizing a monochromatic Al Kaelectrode at 15 kV and 150 W. The coupons were dried and stored in a N_2_ atmosphere before XPS analysis

Coupons used for SEM observation and XPS analysis were not potentiodynamically polarized, so the surfaces were not destroyed.

## Results and Discussion

### Effect of CP on SRB metabolic activity

**Fig 3:**
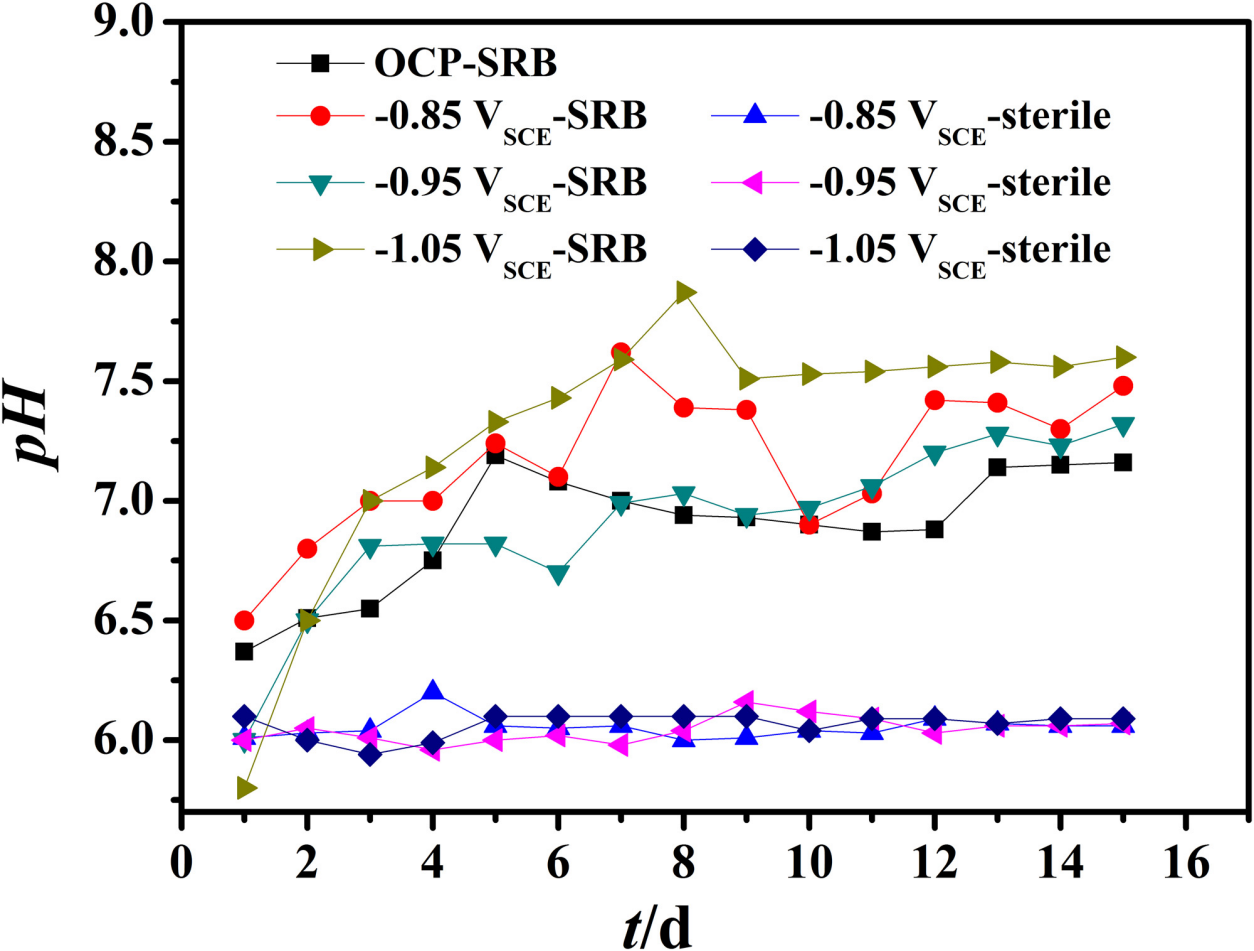
The variation of pH of systems containing SRB with different polarization potentials.

The influence of the cathodic polarization on pH during the experiment period is shown in Fig 3. The pH increased with time as it is polarized to more negative values. The pH of the systems increased by 0.6~1.6versus the initial values with the lowest increase in natural potential while the highest increase was observed at -1.05 V_SCE_. The pH value also increased from 7.1 to 7.6 when the polarization potential shifted negatively from natural potential to -1.05 V_SCE_. This behavior could be explained by electron pathways of DET or MET between SRB and polarized electrode (Fig 1). As the protection potential shifted negatively, the reaction of H^+^ to H accelerated and the consumption of H^+^ and reduction of sulfates resulted in pH increases with time. The experimental results of Zhu [33] showed that hydrogen permeation current density increased rapidly when the cathodic potential was added to the three-electrode system of the cathodic cell, and then the hydrogen permeation current density could slowly obtain a stable value.

To determine whether the increase in pH was caused by SRB or cathodic polarization, we measured the pH change of EQ70 in abiotic media. As the pH in the abiotic media remained stable during the immersion period, the increase of pH in Fig 3 was considered to be caused by SRB metabolic activity.

**Fig 4:**
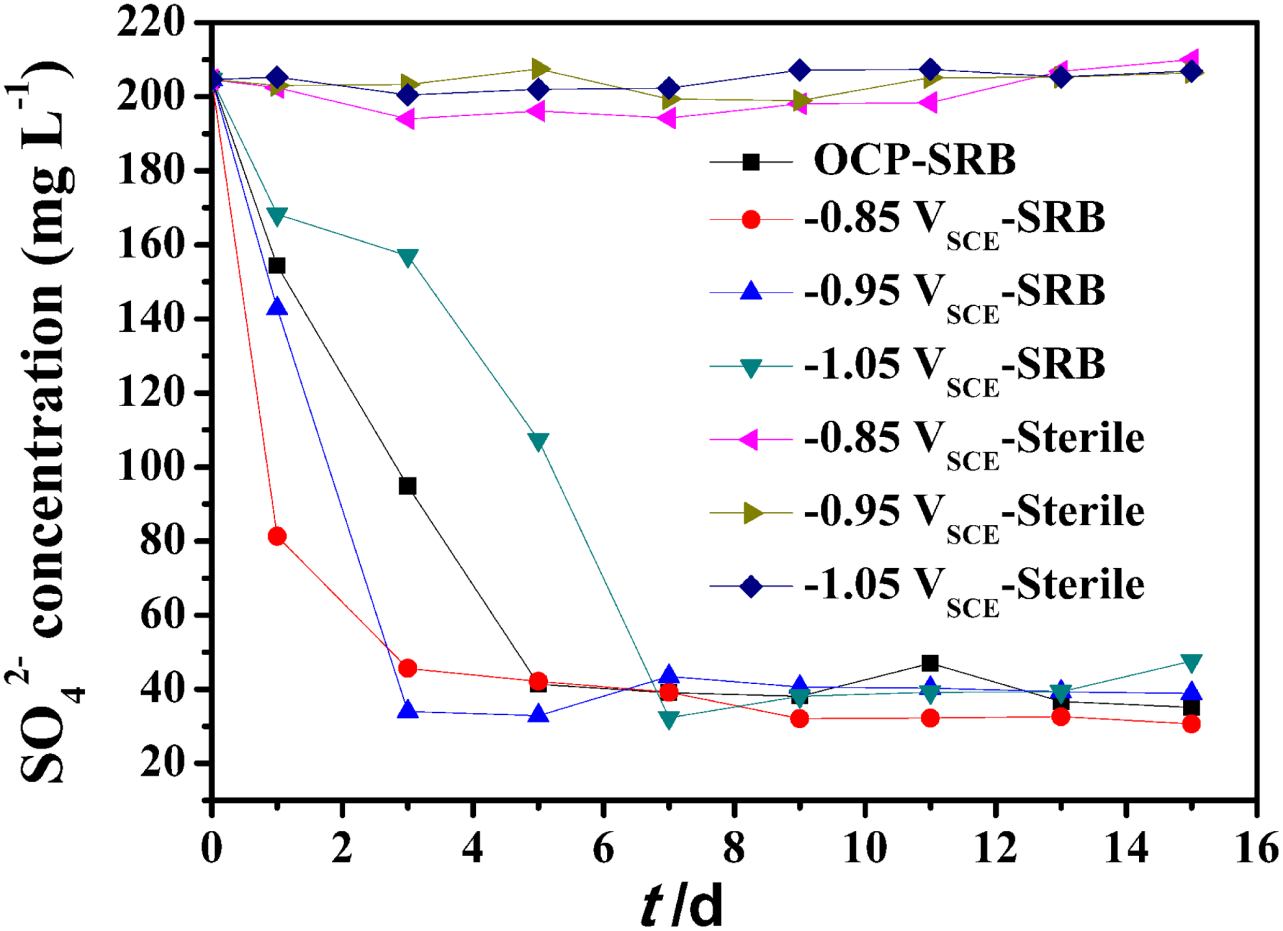
Sulfate variation with time upon different cathodic polarization potential.

The influence of the cathodic polarization potential on SRB metabolic activity in the system is shown in Fig 4. As SRB take sulfate as electron acceptor in metabolic process, the concentration of SO_4_^2-^ could reflect the metabolism activity of SRB indirectly. During the first 7 days, the concentration of SO_4_^2-^ decreased most sharply under -0.85 V_SCE_ CP, while at-1.05 V_SCE_ CP, the concentration of SO_4_^2-^ decreased slower than the system without any applied potential. This means that the conventionally polarized (-0.85 V_SCE_) electrode could promote the metabolic activity of SRB [34], while -1.05 V_SCE_ inhibited it. After 7 days, the concentration of SO_4_^2-^ sulfate ions did not change much, indicating SRB metabolic activity went into decline [35].

Previous studies showed that electrons can be obtained from traditionally polarized electrodes [11, 36, 37]. Thus, the possible metabolic pathways of SRB under -0.85 V_SCE_ CP is that SRB obtains electrons from polarized electrode to reduce sulfates for growth directly or indirectly (Fig 1). However, as polarized potential shifted negatively, the increasing pH and alkaline environment became unsuitable for SRB growth. Studies [26, 38, 39] have demonstrated that a potential more negative than-1.024 V_SCE_ was effective in decreasing bacterial content by 1 to 2 orders of magnitude. So SRB metabolic activity was restricted in CP of -1.05 V_SCE_ (Fig 4). This behavior has been confirmed by previous studies [39]. In these studies, pH increased as the steelwas polarized to negative values, which decreases bacterial growth. At more negative potentials, the pH became alkaline and SRB counts decreased [10].

### Corrosion behavior of EQ70 in SRB medium with different polarization potential

#### Potentiodynamic polarization analysis

If the anodic dissolution process of protected metal follows the Tafel rule, it may be assumed that only one electrode reaction is occurring on the electrode surface in the strong polarization zone of the Tafel polarization curve. Assuming that the dynamic mechanism of this reaction does not change from corrosion potential to measured polarized potential, then, the dynamic mechanism of this reaction can be studied by the following expressions. The relationship between cathodic protection potential *E*_*pr*_ and the anodic dissolution current *I*_*a*_ of metal at *E*_*pr*_ can be expressed as

$$E_{pr}=E_{corr}+\beta_{a}\text{ln}\frac{I_{a}}{I_{corr}}$$ (10)

$$I_{a}=I_{corr}\text{exp}\left[\frac{2.303\times \Delta E}{\beta_{a}}\right]$$ (11)

$$\Delta E=E_{pr}-E_{corr}$$ (12)

$$\beta_{a}=\frac{b_{a}}{\text{ln}10}$$ (13)

Where, the *E*_*corr*_ and *I*_*corr*_ represent the corrosion potential and self corrosion current density of metal without polarization.

**Fig 5:**
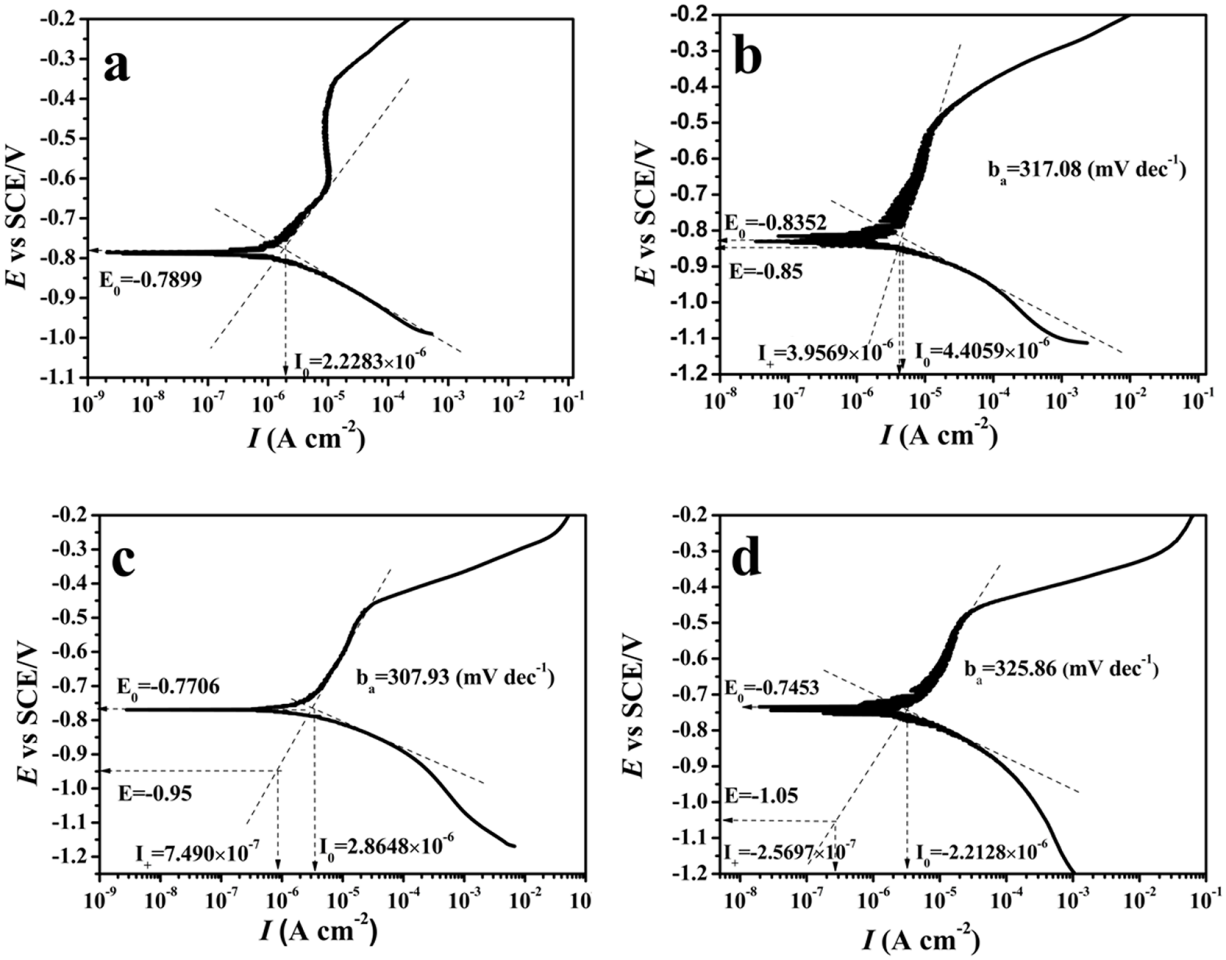
Potentiodynamic polarization curves for EQ70 electrodes in SRB media with different polarization potential.

In the strongly polarized zone, log|*I*_*a*_| is proportional to Δ*E*, *b*_*a*_ and *I*_corr_ can be calculated from a polarized curve, so that *I*_*a*_ at potential *E*_*pr*_ [40] could be calculated by Eq 11 (Fig 5 and Table 1)

**Table 1:** Tafel parameters of the EQ70 specimensexposed to SRB media.

Condition	OCP	-0.85 V_SCE_	-0.95 V_SCE_	-1.05 V_SCE_
*I*_corr_(μA cm^-2^)	2.2283	4.4059	2.8648	2.2128
*E*_corr_ (mV vs SCE)	-789.9	-835.2	-770.6	-745.3
*b*_a_ (mVdec^-1^)	271.48	317.08	307.93	325.86
*I*_*a*_(μAcm^-2^)	2.2280	3.9569	0.7490	0.2570

Fig 5 gives the potentiodynamic polarization curves for EQ70 electrodes in SRB media with different polarization potentials after immersing for 15 days and the results shown in Table 1.*I*_corr_ illustrates the corrosion behavior of steel on the free corrosion potential. The larger *I*_corr_ (4.4059 μA cm^-2^) at -0.85 V_SCE_ than that at OCP (2.228 μA cm^-2^) could be attributed to the enhanced SRB activity, while the smaller *I*_corr_ (2.2128 μA cm^-2^) at -1.05 V_SCE_ than that at OCP (2.228 μA cm^-2^) was caused by the calcareous layer formed on metal surface. However, for -0.95 V_SCE_, although the anodic current *I*_a_ (0.749 μA cm^-2^) was much reduced compared with that without polarization (2.228 μA cm^-2^), *I*_corr_ at -0.95 V_SCE_ is still larger than that at OCP, this means that the CP effect was not valid while CP was removed for the -0.95 V_SCE_ system.

The anodic current (*I*_a_) was significantly different in different systems. Of the four systems investigated, coupons with -0.85 V_SCE_ CP had the highest corrosion current, followed by -0.95 V_SCE_, OCP, and -1.05 V_SCE_. Rather than preventing steel from corrosion, steel under -0.85 V_SCE_ CP had a higher anodic current than that without polarization, which means that -0.85 V_SCE_ CP promoted the corrosion of EQ70 in the SRB medium. The anodic current below -1.05 V_SCE_ was much smaller than that under -0.95 V_SCE_ CP and that at OCP. Although the corrosion current *I*_corr_ at -1.05 V_SCE_ was similar with that under the OCP condition, the anodic current was much smaller. This indicated that coupons at -1.05 V_SCE_ were protected. The difference may be attributed to the enhanced activity of SRB at-0.85 V_SCE_ CP, which is consistent with a recent study [41] that SRB at -0.85 V_CSE_ CP grew better and result in a higher corrosion rate than that at -0.95 V_CSE_ CP. The variations in different cathodic protection effects provide a new way to understand the interaction between SRB and cathodic polarization.

#### Electrical impedance spectroscopy results

At the open circuit potential, there are two reactions—anodic and cathodic. The former is the anodic dissolution process of metals, which is transformed into metal ions. The latter is the reduction of the cathodic depolarizer. The depolarizer can be anything that will be reduced and form an electrochemical couple with the metal electrode. According to electrochemical theory [42], the relationship can be shown as follows:

$$\frac{1}{R_{ct}}=\frac{1}{R_{t,a}}+\frac{1}{R_{t,c}}$$ (14)

Here, *R*_*ct*_, *R*_*t*,*a*_ and *R*_*t*,*c*_ refer to the charge transfer resistance of the electrode reaction, anodic reaction and cathodic reaction, respectively.

EIS has been widely used to study the electrochemical processes that occur at the metal/solution interface. Under CP conditions, the electrode will undergo cathodic polarization due to the impressed current, and the characteristic between metal and biofilm changes accordingly. Over a large range of potential values, the protection effect will be different at different potentials. The value of *R*_*t*,*a*_ increases with the negative cathodic potential while the *R*_*t*,*c*_ decreases at a slow pace. When the electrode potential reaches a certain potential, the *R*_*t*,a_ becomes too large and it can be neglected in Eq 14, the *R*_*t*,_ can be equal to *R*_*t*,*c*_, suggesting that *R*_*t*,*a*_ is hardly reflected in *R*_*t*._

**Fig 6:**
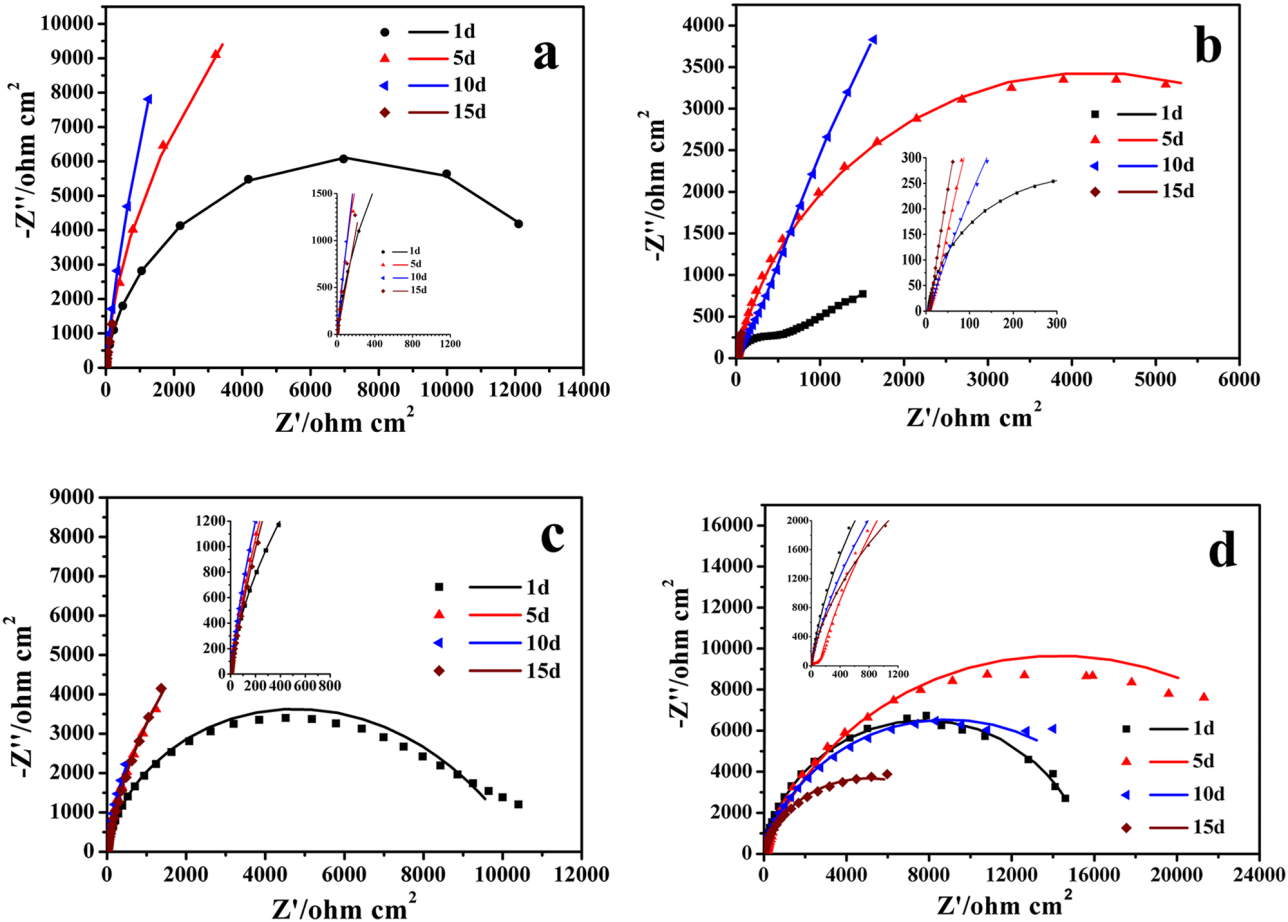
EIS for EQ70 in SRB media as function of the time after polarization at different potentials. a) OCP; b) -0.85 V_SCE_; c) -0.95 V_SCE_; and d) -1.05 V_SCE_.

**Fig 7:**
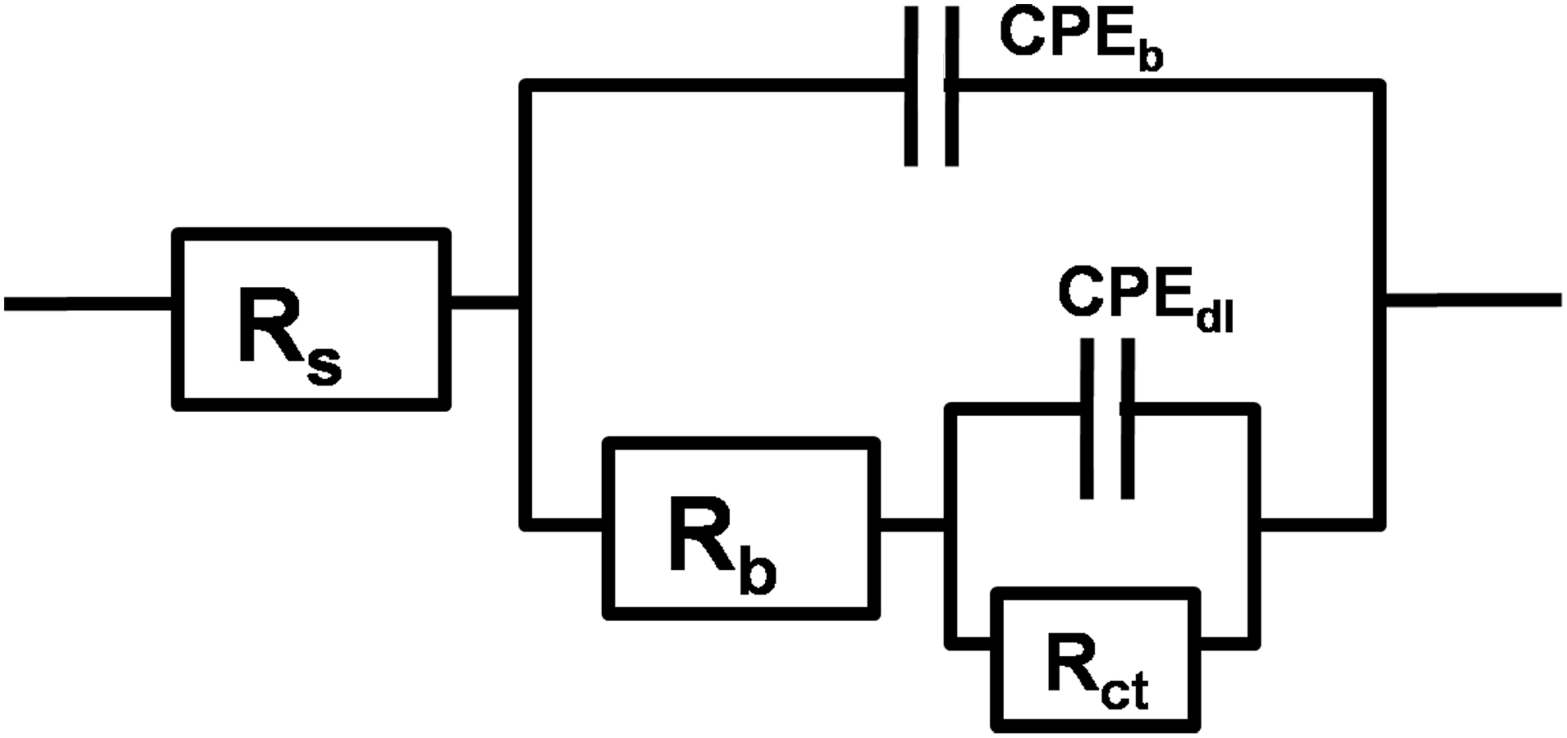
Equivalent circuit used in the analysis of the impedance diagrams in Fig 6.

Given the above analysis, EIS measurements in this study were conducted on a steady-state open circuit potential to find the surface state after polarization. Frequency-dependent impedance measurements were performed at steady-state open circuit potential after polarization for 15 days at different potentials (Fig 6), in which experimental data (scatter dot) were consistent with the best fitline (straight line). The equivalent circuit that was found to reproduce most closely the impendence diagram is shown in Fig 7. The curves were fitted using ZSimpwin software, with chi-square (χ^2^) valueless than 0.01.

The change in *R*_ct_ observed from the analysis of the electrode impedances reflected the corrosion sensitivity of EQ70 (Table 2). On the first day, *R*_ct_ increases with negative potential, this means that the calcification effect outstrips the SRB corrosive impact. With progressing time, the SRB number was increasing in solution, EIS was the multifactor functioning result of microorganism and CP.

**Table 2:** The *R*_c,t_ (ohmcm^2^) values obtained from analysis of the electrode impedances.

t/day	OCP	-0.85 V_SCE_	-0.95 V_SCE_	-1.05 V_SCE_
1	1460	7397	10570	15550
5	13150	8461	20920	29560
10	31020	24240	19480	20200
15	10780	6268	22430	29290

The *R*_ct_ for -0.85 V_SCE_ was smaller than that at OCP after 5 days, the decreased *R*_ct_ value being caused by the enhanced SRB activity at -0.85 V_SCE_; at the -0.95 V_SCE_, the *R*_ct_ was increasing unless at 10^th^ day.

On the contrary, the *R*_ct_ at -1.05 V_SCE_ increased with time, which means that the -1.05 V_SCE_ effectively slowed down the corrosion rate of EQ70 in SRB media. This was attributed to the calcium magnesium deposit formed on the EQ70 surface due to the large cathodic potential. However, the *R*_ct_ for -0.95 V_SCE_ were relatively volatile. On the 15^th^ day, it was smaller than that for -1.05 V_SCE_ and larger than that for -0.85 V_SCE_.

In these circumstances, the anodic and cathodic reactions occurring on the electrode surface share the same potential. The current density is inversely proportional to resistance.

$$\frac{R_{t,a}}{R_{t,c}}=\frac{\left|I_{t,c}\right|}{\left|I_{t,a}\right|}$$ (15)

Here, the *I*_t,a_ and *I*_t,c_ stand for the current density of the anodic reaction and cathodic reaction respectively. The *R*_t,a_ can be calculated by Eq 14 and expressed as followed:

$$R_{t,a}=\left(\frac{\left|I_{t,c}\right|+\left|I_{t,a}\right|}{\left|I_{t,a}\right|}\right)R_{ct}$$ (16)

Here, the *R*_ct_ and *R*_t,a_ refer to the charge transfer resistance of electrode reaction and anodic reaction respectively at cathodic polarization.

For the cathodic reaction, the rate determining process that takes place on the electrode surface is different for different depolarizers. Dissolved oxygen and H_3_O^+^ ions are the main depolarizers in seawater. The choice of which depolarizers happens depends on the concentration of depolarizers and polarized potential [28].

$$\frac{1}{R_{t,c}}=\frac{1}{R_{c,O}}+\frac{1}{R_{c,H}}$$ (17)

When the depolarizer is dissolved oxygen, the key step of cathodic reaction is the activation of oxygen. Increasing the negative potential makes the diffusion process of dissolved oxygen play a more important role in the cathodic half-cell reaction. At the optimum protection potential, the key step is the diffusion of dissolved oxygen while *R*_ct_ reaches its maximum. By increasing the negative potential, the hydrogen depolarization will occur beside the reaction of oxygen [43]. However, this application of EIS to analyze the OCPP is based on the principle that the electrode reaction occurred in the oxygen-rich environment [44]. Here, in this study, the culture solution was sterilized, and the oxygen concentration is low. Thus, this EIS method is not suitable for our present study to determine the OCPP. However, this could help us to understand the dynamic mechanism of the process on the metal/solution surface.

**Fig 8:**
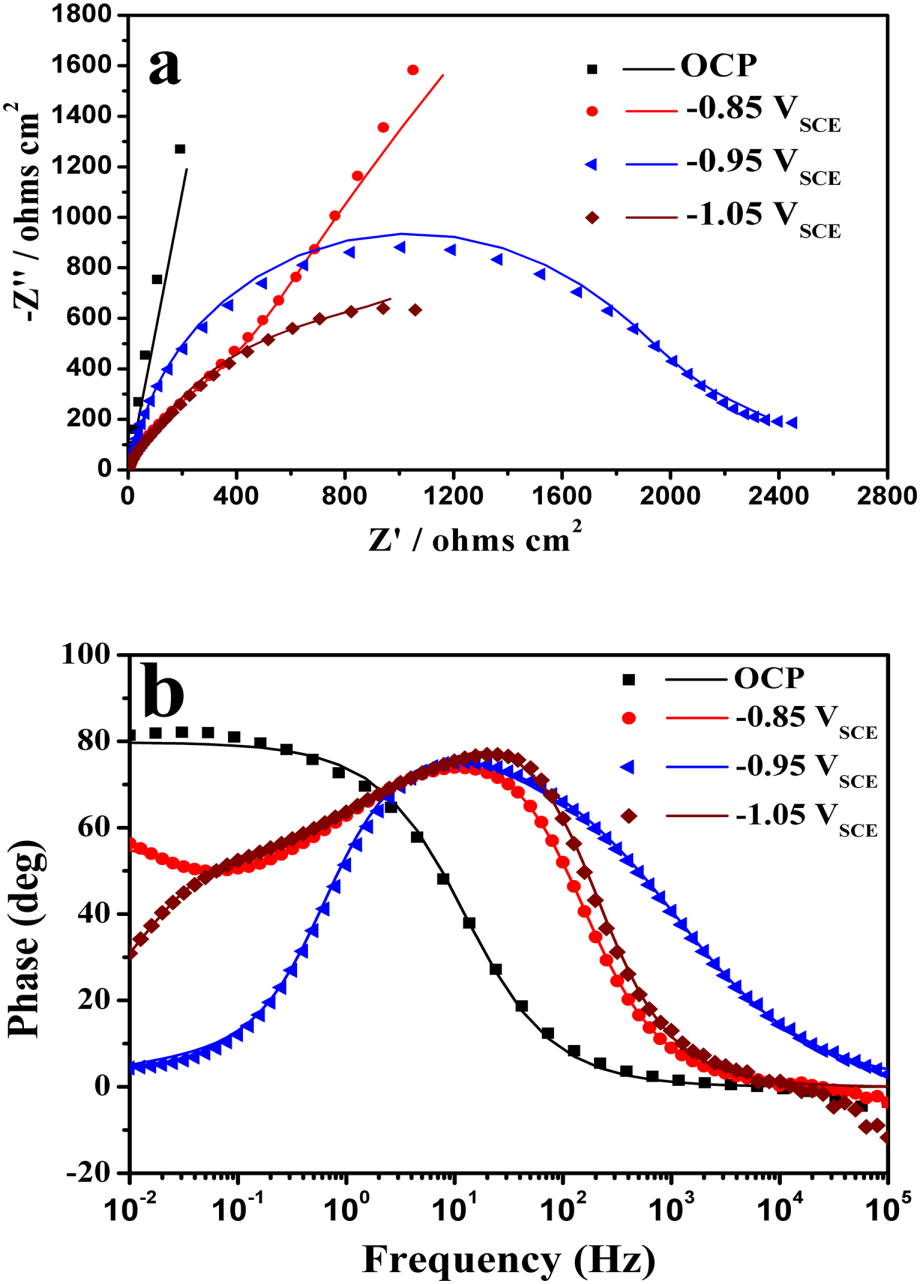
EIS spectra of EQ70 steel at different polarization potentials in SRB medium (a) Nyquist plots and (b) Bode phase-angle plots.

**Fig 9:**
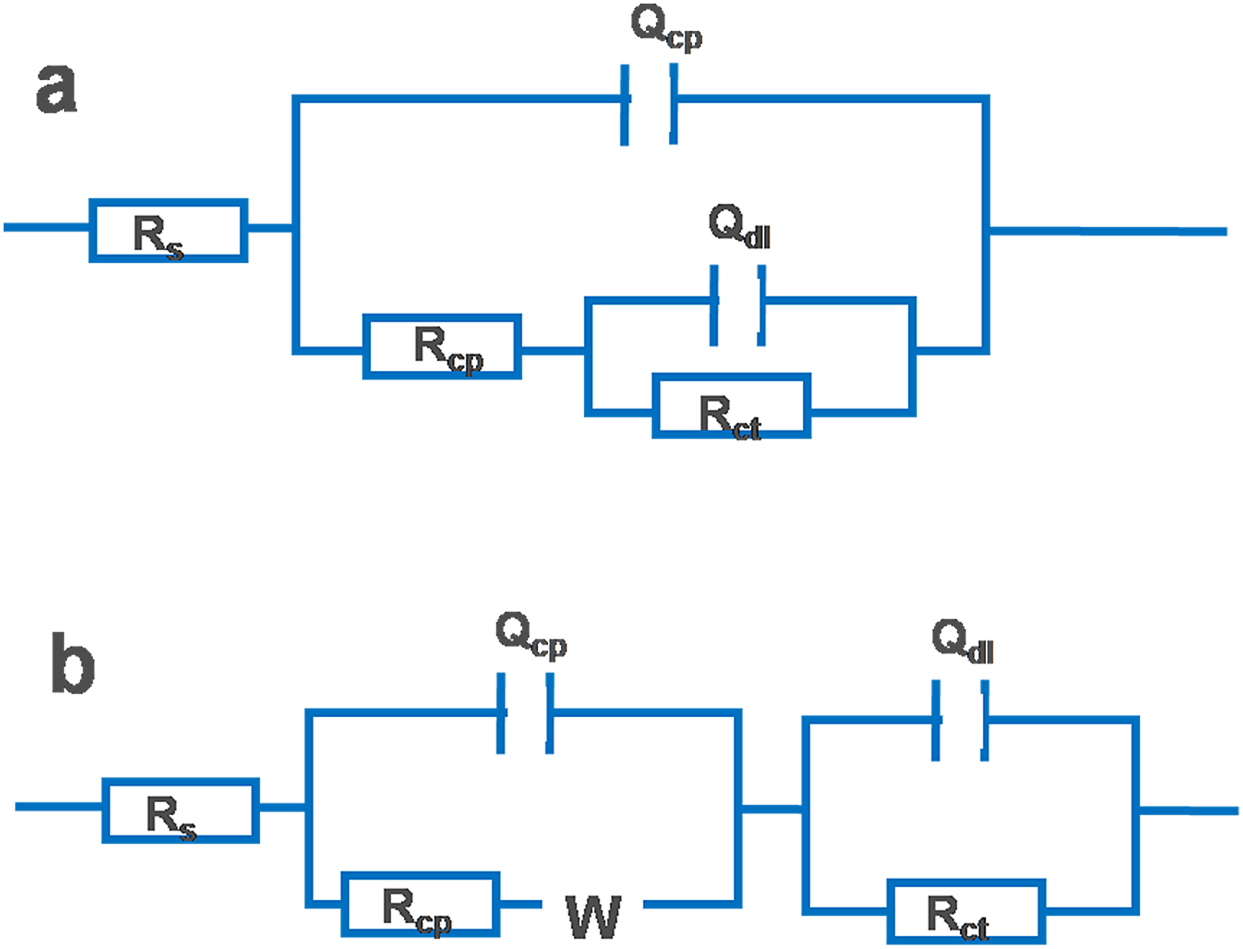
Equivalent circuits used for modeling the impedance spectra of the EQ70 steel specimens exposed to SRB media: (a) model for OCP and -0.85 V_SCE_, (b) model for -0.95 V_SCE_ and -1.05 V_SCE_.

Fig 8 shows the EIS data for various potentials for the samples placed in SRB media for 15 days. Fig 8a shows that impedances were significantly variable at different potentials. The impendence module of the Nyquist semicircles for EQ70 in SRB medium decreased in magnitude with a decrease in the applied potential and Warburg impedances were found at -0.95 V_SCE_ and -1.05 V_SCE_. The phase-angle spectrum obtained at OCP was much different from those with polarization. The circuit representation for the system is shown in Fig 9 [(a) mode for OCP and -0.85 V_SCE_, (b) model for -0.95 V_SCE_ and -1.05 V_SCE_.]

The circuit includes a *R*_s_, which represents the solution resistance, a chargetransfer resistance (*R*_ct_) for the steel specimen surface, a constant-phase element (CPE_dl_) associated with the electrical double layer, and a Warburg element (W) that represents the diffusion process, which is influenced by the polarized potential. The curves were fitted using ZSimpwin software, with a chi-square (χ^2^) value less than 0.01. The *R*_ct_ data analyzed from EIS were calculated by Eq 16 to obtain the anodic current density, which gives insight into the dissolution rate of the metal at the polarization potential. The best fit results are shown in Table 3.

**Table 3:** The *R*_t,a_ values obtained from analysis of the electrode impedances.

Condition	OCP	-0.85 V_SCE_	-0.95 V_SCE_	-1.05 V_SCE_
*R*_ct_(ohmcm^2^)	10780	2600	2337	1595.9
*R*_t,a_(ohmcm^2^)	21560	8828	8.207×10^5^	2.402×10^6^

The magnitude of *R*_ct_ was indicative of the effects of the polarization potential and the SRB. For the potential range employed in this present study, the *R*_ct_ value decreased with the negative potential. The *R*_t,a_ had the minimum value at -0.85 V_SCE_. It then began to increase with a decrease in the potential. This suggested that the metal had higher anodic current density than that at OCP, but a lower metal dissolution rate at -0.95 V_SCE_ and -1.05 V_SCE_.

### The effect of varying cathodic polarization on the surface morphology of EQ70

**Fig 10:**
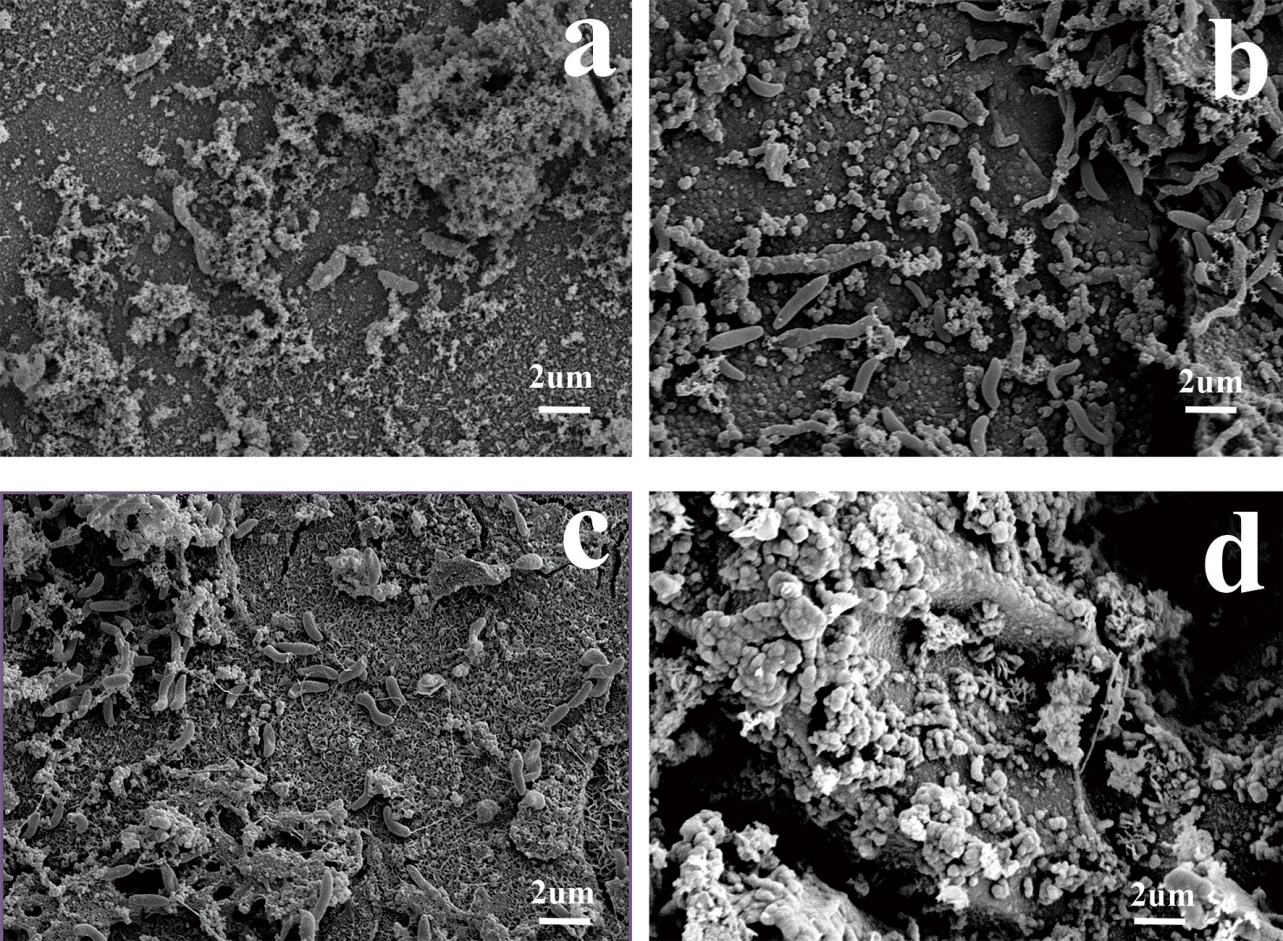
Corrosion surface morphologies of the EQ70 steel at various cathodic polarization potentials in the PGC medium. a) OCP; b) -0.85 V_SCE_; c) -0.95 V_SCE_; d) -1.05 V_SCE_.

Fig 10 shows the surface morphology of the EQ70 steel at various cathodic polarization potentials in the PGC medium with SRB after 15 days. At the natural potential, thick biofilm formed on the surface of theEQ70was observed. At -0.85 V_SCE_ (Fig 10b) and -0.95 V_SCE_ (Fig 10c), there were obvious corrosion cracks on the surface. However, the corrosion morphology of EQ70 with -1.05 V_SCE_ (Fig 10d) was notably different from those under other CP conditions. Corrosion products like calcareous deposits formed on the surface of EQ70, and no bacteria was observed on the surface.

In the presence of iron, SRB vigorously metabolize and produce large quantities of EPS, which rapidly adhere to metal surfaces and form a dense biofilm (Fig 10a). If the protection potential was insufficient, then the cathodic polarization potential could stimulate SRB growth and further corrosion. Thus, obvious corrosion cracks were observed (Fig 10b).

As the potential moved from -0.85 V_SCE_ to -0.95 V_SCE_, the corrosion rate of the metal decreased with the negative cathodic polarization potential, however it was still insufficient to prevent the corrosive attack from SRB and some corrosive cracks were observed on the surface (Fig 10c).

Under the CP of -1.05 V_SCE_, few SRB were found on the EQ70 surface and calcareous-like deposits were formed (Fig 10d). Along with the restrained metabolism of SRB under -1.05 V_SCE_ CP, EQ70 got effectively protected.

**Fig 11:**
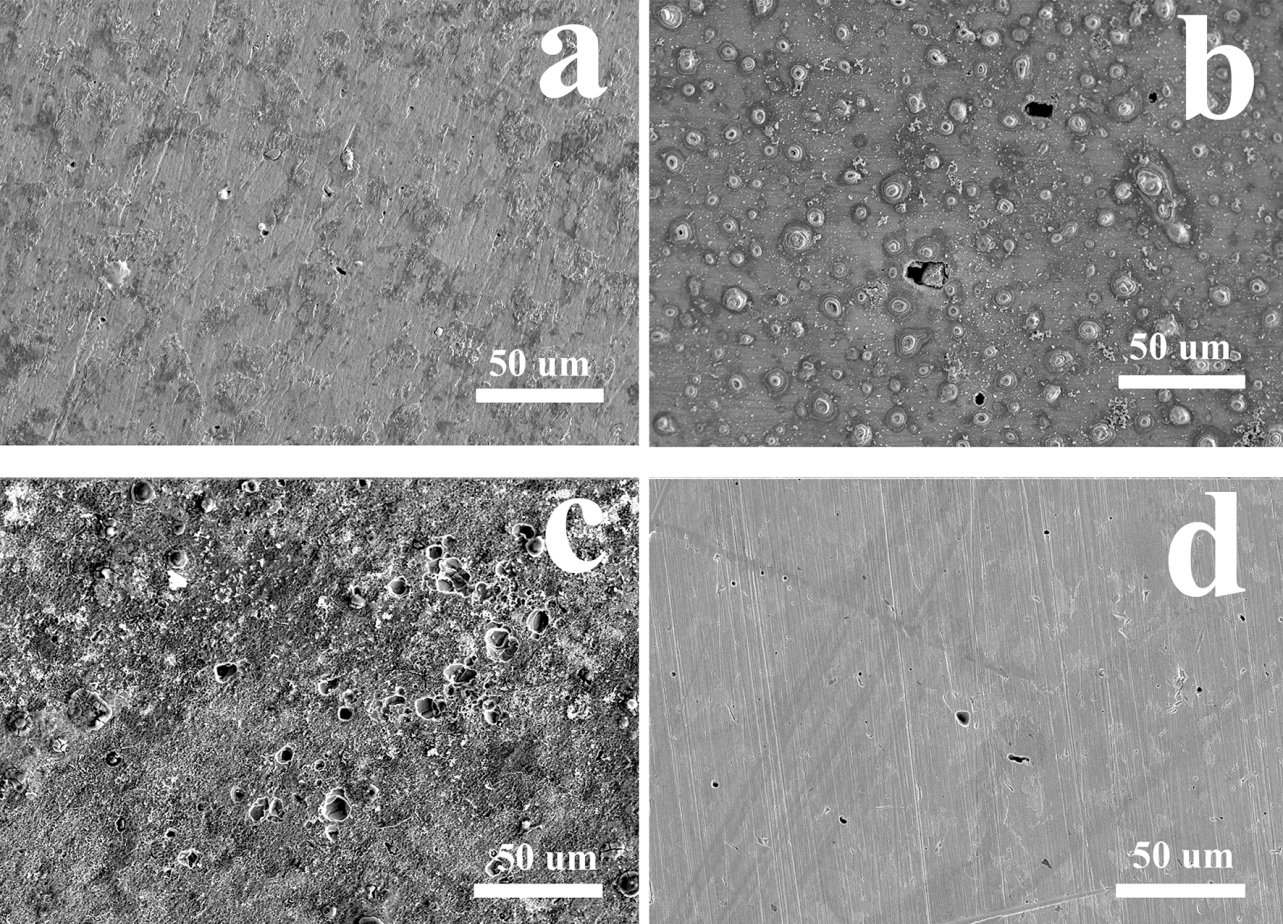
Surface morphologies of the EQ70 steel after removing the corrosion product at various cathodic polarization potentials in the PGC medium. a) OCP; b) -0.85 V_SCE_; c) -0.95 V_SCE_; d) -1.05 V_SCE_.

Surface morphologies of the EQ70 steel after removing corrosion product were observed (Fig 11). It was apparent that micropits occurred in all media. Compared with the coupons without polarization, the surface morphology of coupons became much more irregular and corrosion pits grew much bigger and deeper under the cathodic polarization of -0.85 V_SCE_. The corrosion pits of coupons with -0.95 V_SCE_ cathodic polarization seemed more rounded but no deeper than those under -0.85 V_SCE_ cathodic polarization. By contrast, the surface morphologies of coupons under -1.05 V_SCE_ CP looked much more flat with few pits. These results were consistent with the electrochemical results that -0.85 V_SCE_ CP resulted in more severe corrosion of EQ70 in SRB containing media than that without CP, while -1.05 V_SCE_ CP inhibited this corrosion behavior.

### The effect cathodic polarization on the corrosion products of EQ70

**Fig 12:**
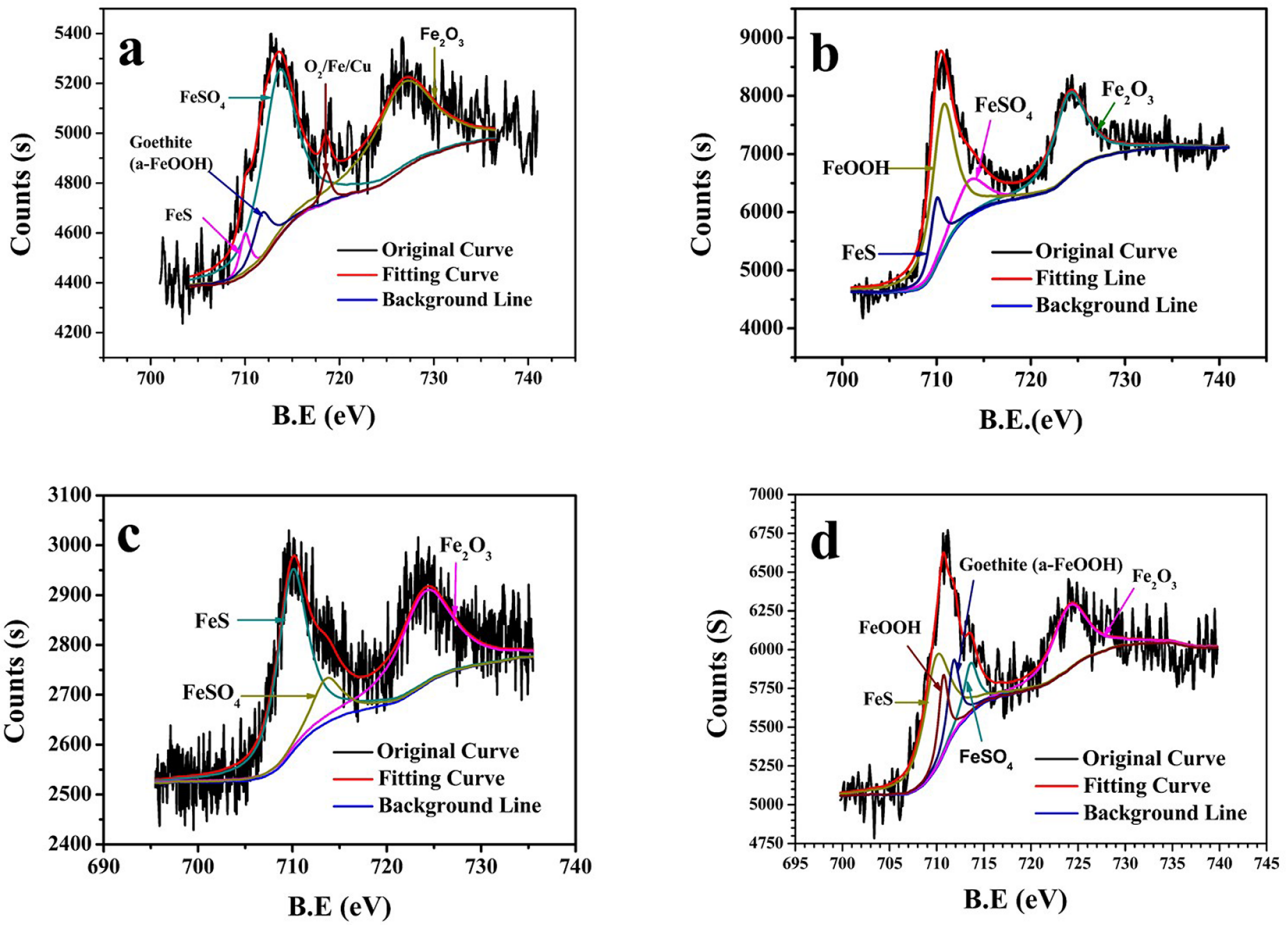
High-resolution Fe spectra of the corrosion product on the steel surface at various cathodic polarization potentials in SRB medium. a) OCP; b) -0.85 V_SCE_; c) -0.95 V_SCE_; d) -1.05 V_SCE_.

XPS analysis was employed to distinguish the differences of corrosion products on EQ70 coupons exposed to SRB media at various cathodic polarization potentials. The coupons were dried and stored in a N_2_ atmosphere prior to XPS analysis. For Fe spectra of the corrosion product, FeS, FeSO_4_, and Fe_2_O_3_ were detected in all the coupons but in varying proportions. The high concentration of sulfide in solution contributed to the large amount of FeS and FeSO_4_ observed on the surface of EQ70 coupons. However, oxidization due to oxygen in the environment and the readily-oxidized property of Fe(II) compounds formed at room temperature. This results in the formation of iron oxides such asFe_2_O_3_ and FeOOH, with variations of those iron oxides between different coupons. A low O_2_/Fe/Cu peak (B.E at 718.6 eV) was only observed on the surface of EQ70 without polarization (Fig 12), which implied that the layer of iron sulfides was rather thin.

For OCP, the Fe2p spectrum consisted of dominant peaks of FeSO_4_ (B.E at 713.6 eV) and Fe_2_O_3_ (B.E at 724.1 eV). When applying the cathodic polarization, O_2_/Fe/Cu peak (B.E at 718.6 eV) disappeared and the percentage of FeS (B.E at 710 eV)in Fe compounds increased while FeSO_4_ decreased (Table 4). The reason may be that, due to the interaction between SRB and the outer potential, the corrosion of EQ70 with cathodic potential of -0.85 V_SCE_ and -0.95 V_SCE_ in SRB media was more severe than that without outer potential. Although the outer potential of -1.05 V_SCE_ provided relatively effective protection for EQ70, the larger cathodic polarization resulted in some calcium magnesium deposition on the surface (Fig 10d). Thus, no Fe peaks were detected in Fig 10d.

**Table 4:** Results of XPS for different iron-positions on the specimen surface at different potentials in SRB medium. (%).

Elements	OCP	-0.85 V_SCE_	-0.95 V_SCE_	-1.05V_SCE_
FeSO_4_	45.88	13.79	11.20	9.65
FeS	3.48	10.78	46.61	38.67
Fe_2_O_3_	41.67	33.51	42.19	29.74
Goethite(a-FeOOH)	6.66	-	-	11.66
FeOOH	-	41.92	-	10.26
Fe	2.30	-	-	-

**Fig 13:**
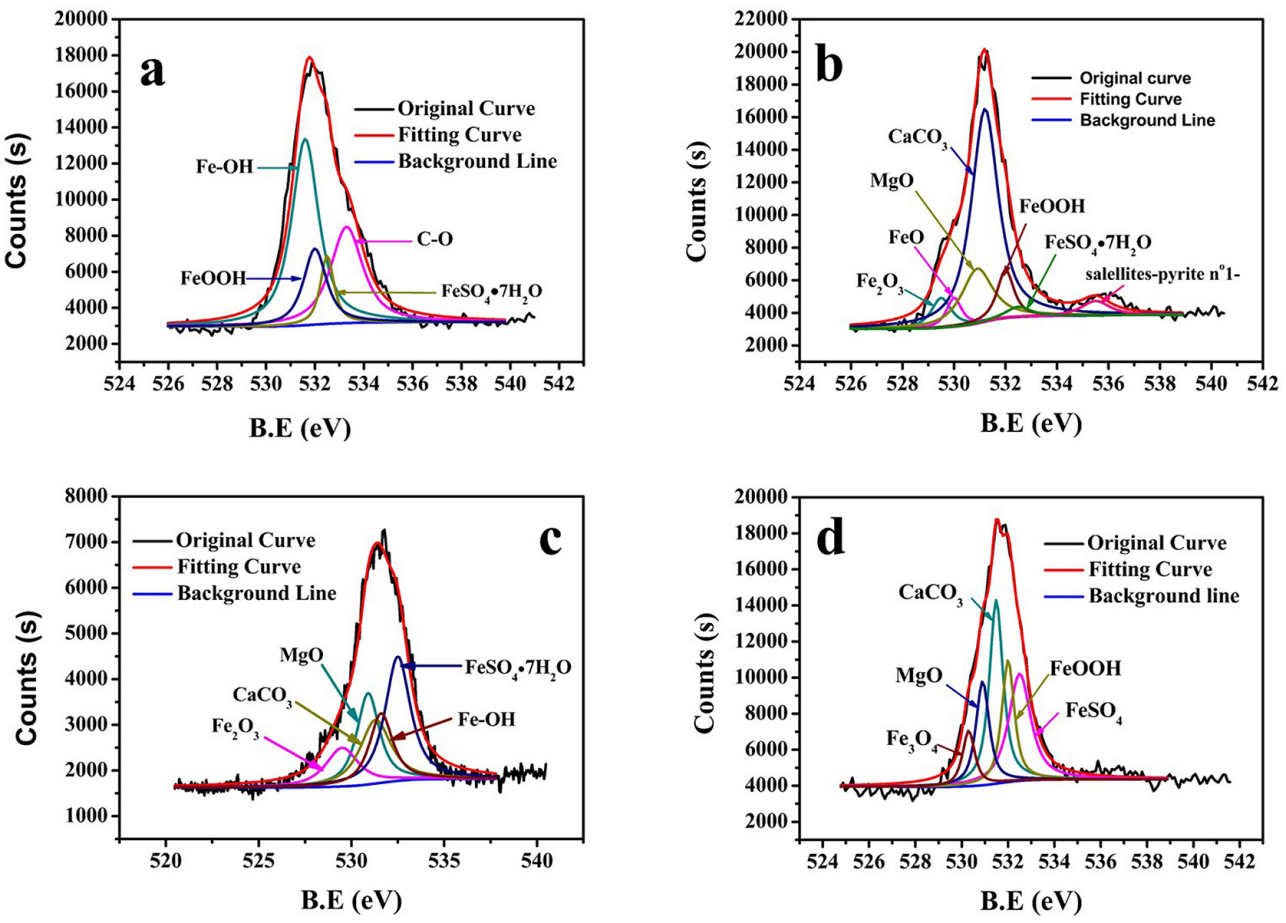
High-resolution S spectra of the corrosion product on the steel surface at various cathodic polarization potentials in SRB medium. a) OCP; b) -0.85V_SCE_; c) -0.95V_SCE_; d) -1.05V_SCE_.

The sulfur-containing compounds formed on the surface layer of the corrosion product are similar among different treatments. The S spectrum had dominant peak components corresponding to FeS_2_, Na_2_SO_4_, CaSO_4_, and FeSO_4_ (Fig 13). However, a larger proportion of less-negative S (S_2_O_3_^2-^, S_n_^2-^, MnPS_3_, pyrrhotite Fe_0.89_S) were found under cathodic polarization. The iron sulfide precipitation is being cathodically reduced just below a potential of -0.1 V_SCE_ [22]. Peaks in lower binding energy were broader and higher after cathodic polarization implying that more sulfites were reduced in those conditions.

**Fig 14:**
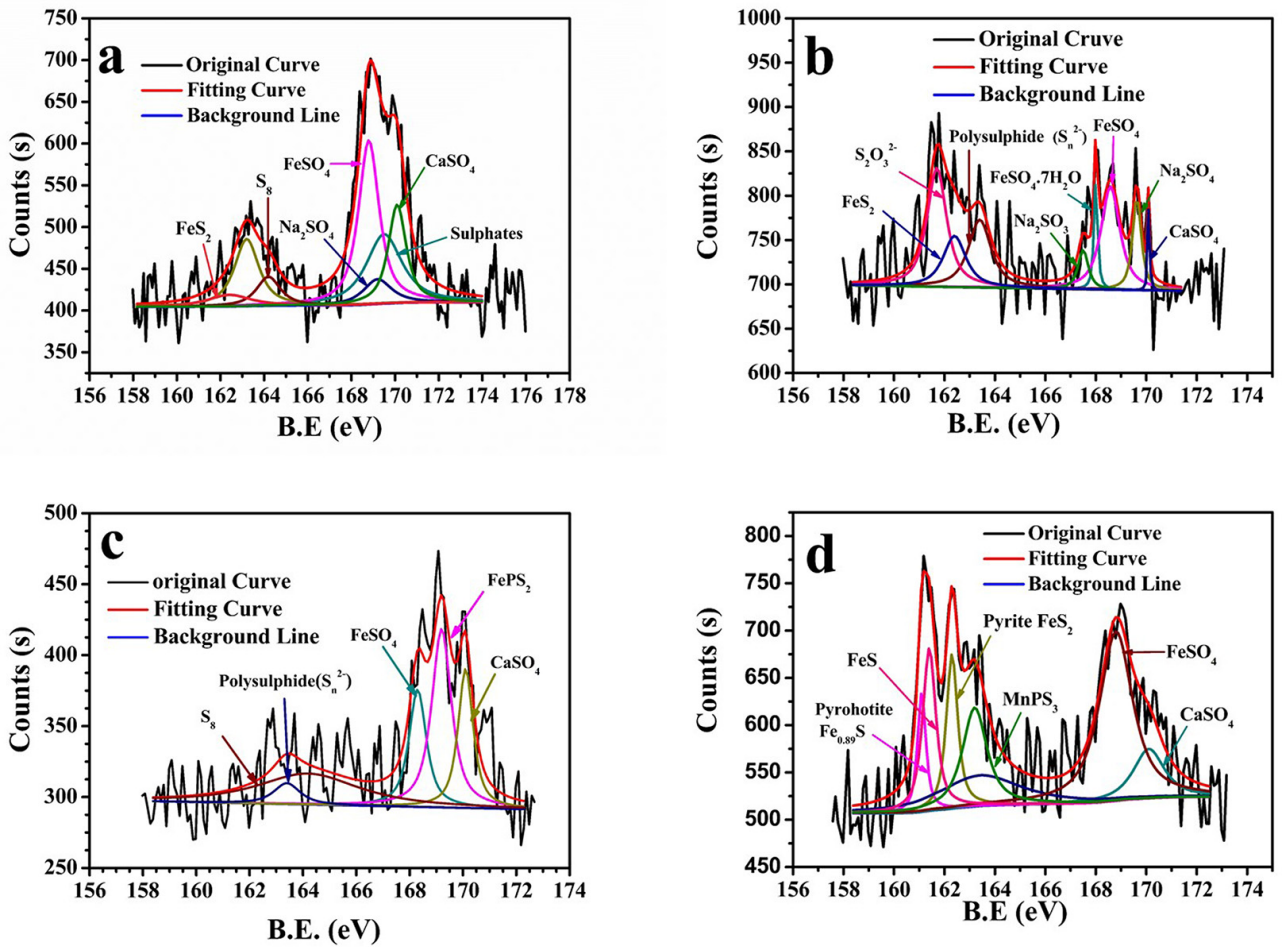
High-resolution O spectra of the corrosion product on the steel surface at various cathodic polarization potentials in SRB medium. a) OCP; b) -0.85V_SCE_; c) -0.95V_SCE_; d) -1.05V_SCE_.

The appearance of oxide may be related with the pH increase in solution and the unavoidable oxygen in the experiment. The C-O compound in the O spectra may be related to the bacteria and the EPS (Fig 14). Calcium and magnesium oxides (MgO, CaCO_3_) were found after applying cathodic polarization. The application of cathodic protection led to the transformation of sulfide rusts into carbonates rusts [45].

The reaction can be written as:

$$Fe_{4}^{\text{II}}Fe_{2}^{\text{III}}{\left(OH\right)}_{12}SO_{4}•8H_{2}O+HCO_{3}^{-}\to Fe_{4}^{\text{II}}Fe_{2}^{\text{III}}{\left(OH\right)}_{12}CO_{3}•2H_{2}O+6H_{2}O+SO_{4}^{2-}+H^{+}$$

or:

$$Fe_{4}^{\text{II}}Fe_{2}^{\text{III}}{\left(OH\right)}_{12}SO_{4}•8H_{2}O+CO_{3}^{2-}\to Fe_{4}^{\text{II}}Fe_{2}^{\text{III}}{\left(OH\right)}_{12}CO_{3}•2H_{2}O+6H_{2}O+SO_{4}^{2-}$$

Also other reactions caused an increase in alkalinity that forced a shift in the equilibria of all chemical reactions involving calcium, magnesium, and bicarbonate irons[46]:

$$HCO_{3}^{-}+OH^{-}\to H_{2}O+CO_{3}^{2-}$$

$$Ca^{2+}+CO_{3}^{2-}\to CaCO_{3}$$

$$Mg^{2+}+2OH^{-}\to Mg{\left(OH\right)}_{2}$$

### SRB and cathodic polarization

In this study, the applied potentials were OCP, -0.85 V_SCE_, -0.95 V_SCE_, and -1.05 V_SCE_. The reaction of H^+^ to H accelerates as protection potential shift negatively. The SRB metabolic activity was promoted in -0.85 V_SCE_ and -0.95 V_SCE_ versus OCP. If the increase in pH was caused by the consumption of H by SRB as shown in Eq 6, then SRB metabolic activity should be at accelerated but not restrained in -1.05 V_SCE_—however, this was exactly the opposite of what was found. The pH at -1.05 V_SCE_ was higher than other systems. The higher pH resulted from the accelerated H production rate leading to redundant OH^-^. Although some SRB were expected to be efficient oxidizers of hydrogen [47], there have also been studies suggesting more direct access to electrons from iron than via hydrogen consumption [48]. Hendrik [49]conducted studies with corrosive SRB and found direct consumption of iron-derived electrons rather than of H_2_ as a crucial mechanism. They found that corrosive SRB significantly stimulated the cathodic reaction, while non-corrosive yet H_2_-consuming control SRB had no effect.

In the study by Miriam [17], the electron transfer mechanisms using cathodes as electron donors for microbial metabolism were elaborated in details. Some bacteria are capable of transferring metabolic electrons through a chain of c-type cytochromes across the cell envelope to extracellular electron acceptors. Electron transfer components of *G*.*sulfurreducens* were not affected by applied anode potential [50].

If SRB could get electrons from proper cathodic polarized electrode more easily than other electron pathways, then it is possible that this electron transfer occurs directly. In the study of Hana [51], the enhanced capacity of *G*. *sulfurreducens strain* KN400 than *G*. *sulfurreducens strain DL-1* on graphite anode poised at -0.4 V_Ag/AgCl_ was associated with a greater abundance of electrically conductive microbial nanowires—even when growing with the soluble electron acceptor. Thus, the enhanced current density was associated with appropriate selective pressure and clear properties of different bacteria [51].

Similarly, in this study, if the SRB strain *Desulfovibrio caledoniensis* could obtain electrons from -0.85 V_SCE_ cathodic polarized electrodes more easily than other electron pathways, then it is possible that this process is achieved by specific protein-based structures or conductive nanowires. The easier electron pathway contributed to the enhanced SRB metabolic activity in -0.85 V_SCE_. The increasing pH can be explained by Eq 9. However, at -1.05 V_SCE_, the formation of calcareous deposits and the alkaline environment on the surface of polarized steel was not suitable for SRB to obtain electrons from the overly negative polarized electrode and growth. Thus, SRB metabolic activity was restrained at-1.05 V_SCE_. Saravia reported a dramatic decrease in bacteria settlement and growth by CP in the initial stages of biofilm formation[52].

In theory, the number of SRB and their metabolic activity was considered sufficient to influence microbiological influenced corrosion. Attached microorganisms on the steel surface would accelerate the process of corrosion. The electron transfer resistance also decreased with the formation of nanowires. Thus, SRB metabolic activity was enhanced and EQ70 had a higher corrosion rate at -0.85 V_SCE_ CP, while at -1.05 V_SCE_ CP, the SRB metabolic activity was suppressed and EQ70 had a lower corrosion rate. The hypothesis of electron pathway has been proposed according to the experimental data and theory analysis based on a summary of previous research, and requires further research.

## Conclusions

The effect of different cathodic polarization potentials on SRB metabolic activity and its influence on EQ70 corrosion were experimentally demonstrated in this study. The results showed that as the potential became more negative, the pH values of the medium increased due to metabolic activity of SRB. The metabolic activity of SRB was increased below -0.85 V_SCE_ CP and restrained under -1.05 V_SCE_ CP. The electrochemical data showed that the cathodic polarization potential of -0.85 V_SCE_ accelerated the corrosion of EQ70 in SRB medium while -1.05 V_SCE_ inhibited the corrosion. Those results were reinforced by SEM and XPS analysis. The enhanced SRB metabolic activity was most probably caused by the direct electron transfer from the electrode polarized at -0.85 V_SCE_, and this direct electron transfer pathway was unavailable in -1.05 V_SCE_.

## Supporting Information

S1 Appendix Duplicate data of sufate variation and EIS data. (DOCX)

S1 Fig Sulfate variation with time upon different cathodic polarization potential. (TIF)

S2 Fig EIS for EQ70 in SRB media as function of the time after polarization at different potentials. a) OCP; b) -0.85 V_SCE_; c) -0.95 V_SCE_; and d) -1.05 V_SCE_.
(TIF)
