# Supplementary material for: Influence of Sulfate-Reducing Bacteria on the Corrosion Behavior of High Strength Steel EQ70 under Cathodic Polarization
Source: PLoS One. 2016 Sep 7;11(9):e0162315. doi: 10.1371/journal.pone.0162315 (PMC5014316; doi:10.1371/journal.pone.0162315)
Supplement: S1 Appendix — (DOCX) [file pone.0162315.s001.docx]

**Influence of sulfate-reducing bacteria on the corrosion behavior of high strength steel EQ70 under cathodic polarization**

Fang Guan, Xiaofan Zhai, Jizhou Duan, Meixia Zhang, Baorong Hou


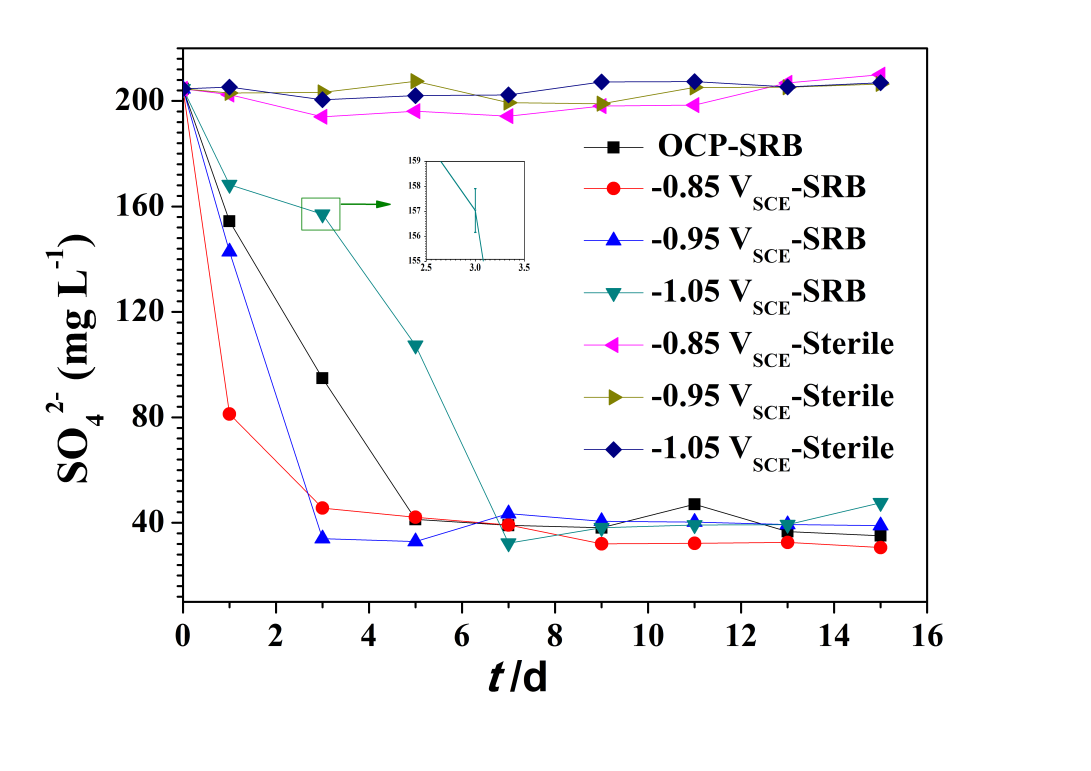


**S1 Fig.** Sulfate variation with time upon different cathodic polarization potential.

While the data showed fairly good consistency, variance was enlarged to be easily recognized in S1 Fig.

During this one month period, we made the duplicated tests of electrical impedance spectroscopy (EIS) measurement using a 10 mV sinusoidal signal with frequencies ranging from 100 kHz to 10 mHz at the steady-state open circuit potentials of coupons, the results were analyzed using Princeton ZSimpWin version 3.21 software. The results were shown in S2 Fig and S1 table.

**
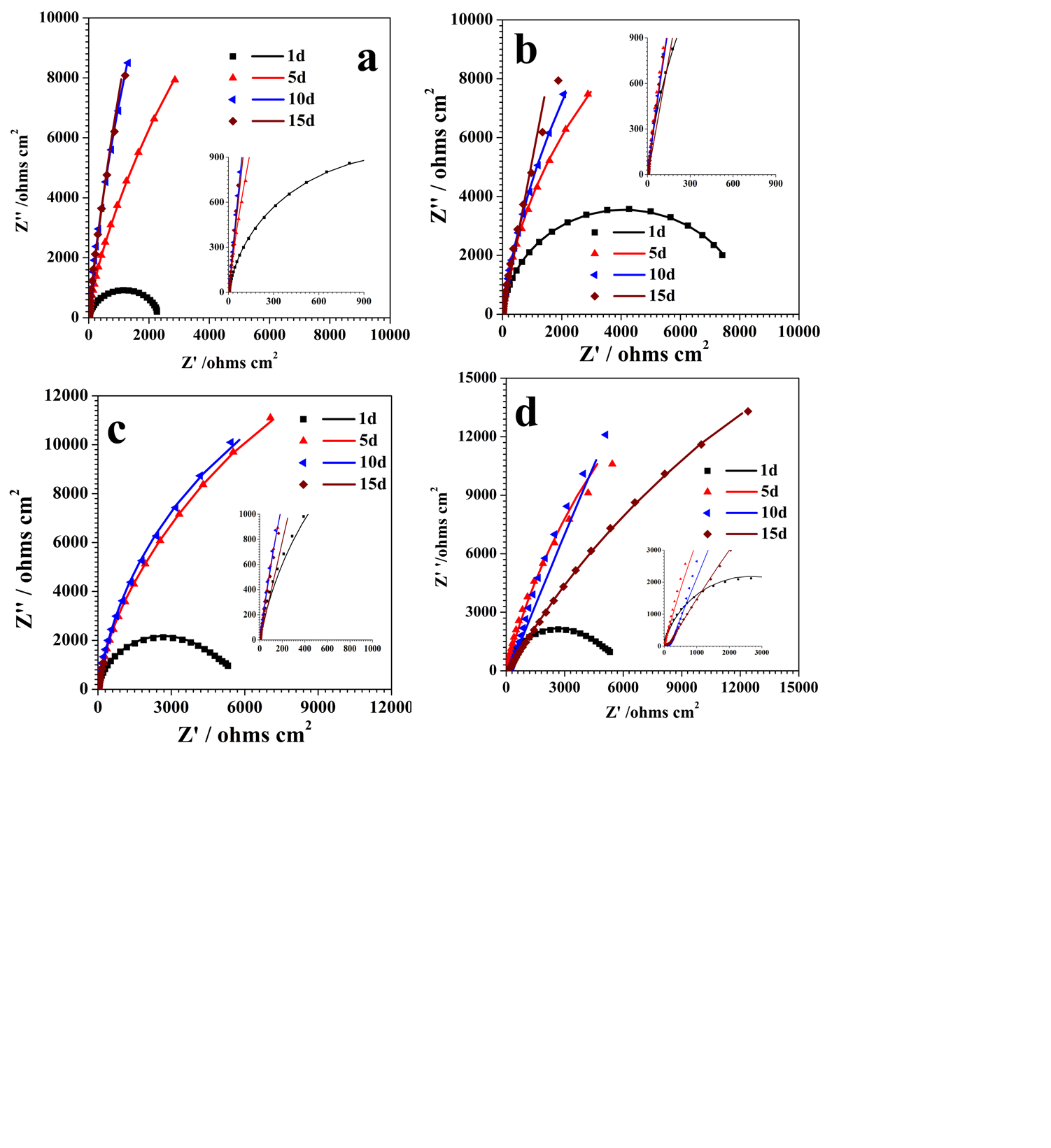
**

**S2 Fig.** EIS for EQ70 in SRB media as function of the time after polarization at different potentials. a) OCP; b) -0.85 V_SCE_; c) -0.95 V_SCE_; and d) -1.05 V_SCE._

Table S1. The *R*_c,t_ (ohm cm^2^) values obtained from analysis of the electrode impedances.

| t/day | OCP | -0.85 V_SCE_ | -0.95 V_SCE_ | -1.05 V_SCE_ |
| --- | --- | --- | --- | --- |
| 1 | 364 | 644 | 788 | 19410 |
| 5 | 9950 | 9050 | 12070 | 68300 |
| 10 | 39268 | 35208 | 53960 | 70070 |
| 15 | 35308 | 31265 | 60270 | 72500 |

On the first day, *R*_c,t_ increased with polarization shifted negatively, this indicated that the calcification effect outstrips the SRB corrosive impact. The *R*_ct_ for -0.85 V_SCE_ was smaller than that at others after immersed in SRB medium for 5 days, which was caused by enhanced SRB activity at -0.85 V_SCE_; while *R*_ct_ increased with time at -1.05 V_SCE_, which indicated that this potential provided protection for coupons in SRB medium. The results showed the same trends with the results shown in manuscript. However, biological activity was much different under different environment, and this could in return influence the electrochemical behavior of specimens, which resulted in the variations in data.
