# Supplementary figures and images for: Influence of Sulfate-Reducing Bacteria on the Corrosion Behavior of High Strength Steel EQ70 under Cathodic Polarization
Source: PLoS One. 2016 Sep 7;11(9):e0162315. doi: 10.1371/journal.pone.0162315 (PMC5014316; doi:10.1371/journal.pone.0162315)

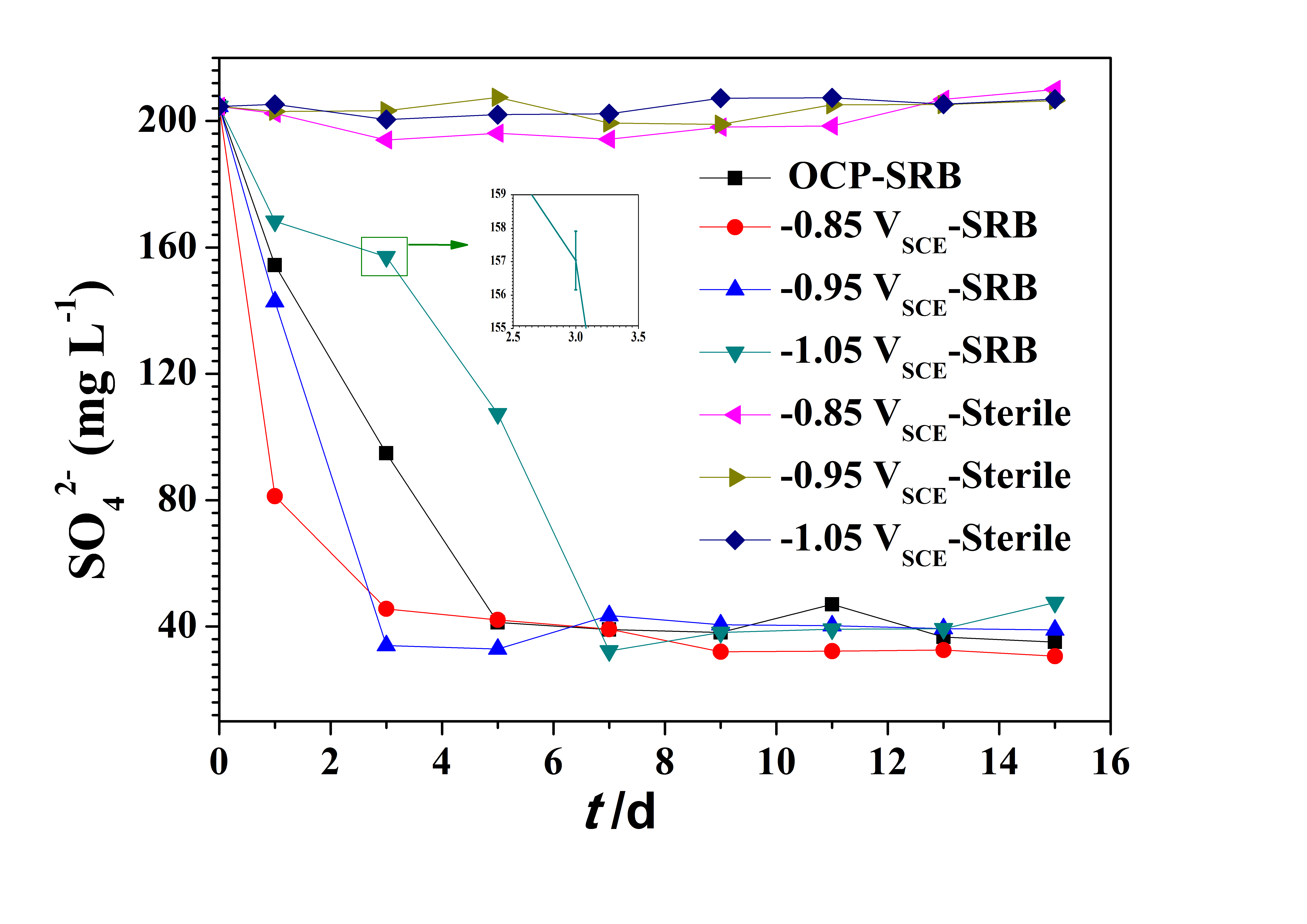

Supplement: S1 Fig — (TIF) [file pone.0162315.s002.tif]

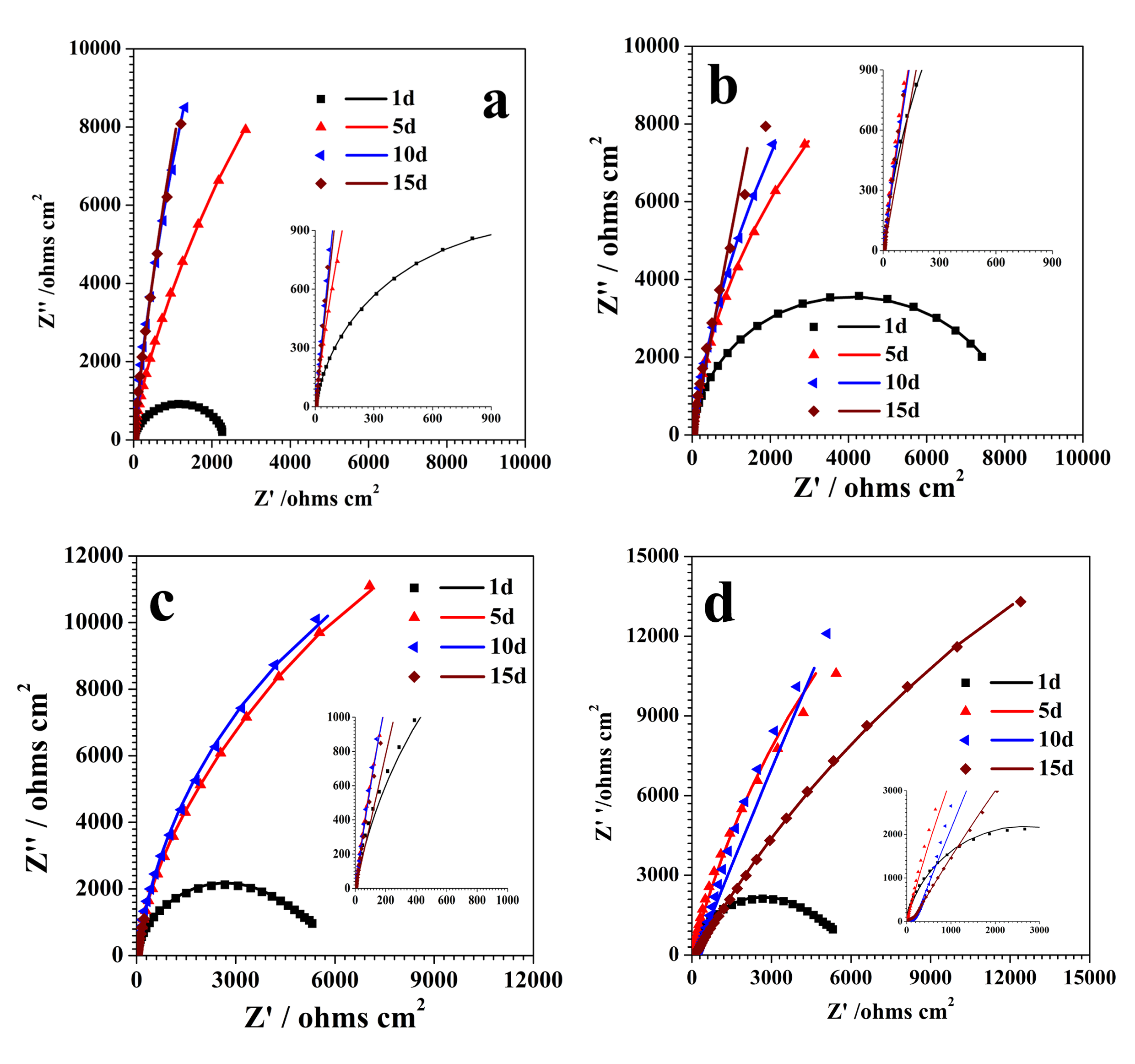

Supplement: S2 Fig — a) OCP; b) -0.85 VSCE; c) -0.95 VSCE; and d) -1.05 VSCE. (TIF) [file pone.0162315.s003.tif]
